# A single point mutation in cyclin T1 eliminates binding to Hexim1, Cdk9 and RNA but not to AFF4 and enforces repression of HIV transcription

**DOI:** 10.1186/1742-4690-11-51

**Published:** 2014-07-01

**Authors:** Alona Kuzmina, Nina Verstraete, Sigal Galker, Maayan Maatook, Olivier Bensaude, Ran Taube

**Affiliations:** 1 The Shraga Segal Department of Microbiology, Immunology and Genetics Faculty of Health Sciences, Ben-Gurion University of the Negev, P.O. Box 653, Beer-Sheva 84105, ISRAEL; 2 Ecole Normale Supérieure, Institut de Biologie de l’ENS (IBENS), and Inserm U1024, and CNRS UMR 8197, Paris F-75005, France

**Keywords:** HIV latency, Transcription repression, Tat, Positive Transcription Elongation Factor b, Super Elongation Complex

## Abstract

**Background:**

Human immunodeficiency virus (HIV) gene expression is primarily regulated at the step of transcription elongation. The viral Tat protein recruits the Positive Transcription Elongation Factor b (P-TEFb) and the Super Elongation Complex (SEC) to the HIV promoter and enhances transcription by host RNA polymerase II.

**Results:**

To map residues in the cyclin box of cyclin T1 that mediate the binding of P-TEFb to its interacting host partners and support HIV transcription, a pool of N-terminal cyclin T1 mutants was generated. Binding and functional assays in cells identified specific positions in cyclin T1 that are important for (i) association of P-TEFb with Hexim1, Cdk9 and SEC/AFF4 (ii) supporting Tat-transactivation in murine cells and (iii) inhibition of basal and Tat-dependent HIV transcription in human cells. Significantly, a unique cyclin T1 mutant where a Valine residue at position 107 was mutated to Glutamate (CycT1-V107E) was identified. CycT1-V107E did not bind to Hexim1 or Cdk9, and also could not assemble on HIV TAR or 7SK-snRNA. However, it bound strongly to AFF4 and its association with HIV Tat was slightly impaired. CycT1-V107E efficiently inhibited HIV replication in human T cell lines and in CD4(+) primary cells, and enforced HIV transcription repression in T cell lines that harbor a transcriptionally silenced integrated provirus.

**Conclusions:**

This study outlines the mechanism by which CycT1-V107E mutant inhibits HIV transcription and enforces viral latency. It defines the importance of N-terminal residues of cyclin T1 in mediating contacts of P-TEFb with its transcription partners, and signifies the requirement of a functional P-TEFb and SEC in mediating HIV transcription.

## Background

Since its discovery, Human Immunodeficiency Virus type 1 (HIV-1), the causative agent of AIDS, has led to the death of close to 25 million people worldwide. Despite the availability of effective anti-HIV therapy (HAART) that successfully limits HIV replication, HAART has failed to completely eradicate HIV. Cell-cell spread permits ongoing viral replication and drug-resistant variants appear. Moreover, once therapy is interrupted, transcriptional silent proviruses re-emerge from infected cell reservoirs and viral replication resumes [1-4].

HIV has become a model for studying transcriptional elongation in eukaryotes, where the viral protein Tat serves as a master regulator of viral transcription. It directly binds to the host cyclin T1 (CycT1) and assembles on the transactivation response RNA element-TAR. Tat thus recruits the Positive Transcription Elongation Factor b (P-TEFb) to the viral promoter and stimulates HIV gene transcription. The Cdk9 kinase subunit of P-TEFb then phosphorylates the C-terminal domain (CTD) of RNA Polymerase II (RNAPII), as well as negative factors, Spt5 and NELF [5-8], to release RNAPII from its pausing and promote viral transcription [7,9-11]. In cells, P-TEFb associates with a second transcription complex - Super Elongation Complex (SEC) [12-14]. Both complexes synergistically act on the same RNAPII and enhance transcription. The ELL2 subunit of SEC increases the catalytic activity of RNAPII by preventing its backtracking and pausing [15]. ELL2 expression is regulated by the E3 ubiquitin ligase, Siah1, which targets the protein for degradation. In infected cells, Tat also associates with SEC, elevating the short half-life of ELL2 and stabilizing P-TEFb-SEC complex formation [12-14,16]. In SEC, AF4/FMR2 family member 4 (AFF4) acts as a scaffold that bridges P-TEFb to SEC [14]. Structural and biochemical studies have shown that AFF4 recognizes P-TEFb through a weak direct interaction with the N-terminus 300 region of CycT1. This interaction is slightly increased when Cdk9 is coupled to CycT1. AFF4 also establishes direct contacts with Tat, which significantly increases the affinity of P-TEFb to SEC. Recently, AFF1 has also been reported to be part of P-TEFb complex [17]. AFF1 increases the affinity of Tat to CycT1 and enhances the release of P-TEFb from its inactive 7SK-snRNP complex to stimulate Tat transactivation and HIV replication. Structural analysis of AFF4/SEC shows it possesses a Tat-binding pocket at the first 300-N-terminal region. Upon AFF4 binding to P-TEFb, Cdk9 undergoes structural changes though is not considered a primary partner of AFF4 [18].

As P-TEFb is essential for HIV replication, its activity is tightly regulated in cells. *In vivo*, there exists equilibrium between active and non-active P-TEFb. The active complex consists of Cdk9 and either cyclin T1, T2a or T2b, while the non-active complex comprises of Cdk9, cyclin T1, and the 7SK snRNP. The latter comprises of a 7SK small nuclear non-coding RNA (snRNA) and the HMBA-inducible protein 1 or 2 (Hexim1/2) [19,20]. *In vivo*, 7SK snRNA forms a core complex with the MePCE and LARP7 proteins [21]. C-terminal residues of Hexim1 mediate binding to CycT1, while C-terminal residues of the cyclin box of CycT1 are important to the interactions with Hexim1 [22-26]. Importantly, Tat and Hexim1 compete for CycT1, as the same Tat-TAR recognition motif (TRM) on CycT1 also binds Hexim1 [26,27]. Nevertheless, we have recently demonstrated that separate binding surfaces on the N-terminal residues of CycT1 are involved in association with Tat or Hexim1 [28].

The structure of P-TEFb, Hexim1 and 7SK RNA have been solved by X-ray crystallography or NMR spectroscopy [18,29-35]. However, little is known on the binding surfaces of P-TEFb/CycT1 that mediate the complex interactions with its transcription partners, mainly AFF4, Hexim1 and Cdk9 and their involvement in mediating HIV transcription. Moreover, the significant of either P-TEFb or SEC to transcription is also not fully understood. Herein, an extensive biochemical analysis was performed on a pool of N-terminal CycT1 mutants that were generated by error prone PCR on the cyclin box of CycT1. CycT1 mutants were analyzed for binding to Hexim1, Cdk9, AFF4 and Tat. The ability of CycT1 mutants to support Tat transactivation in murine and human cells was monitored. A unique V107E mutant of CycT1 was identified. HA-CycT1-V107E did not bind Hexim1, or Cdk9 and its association with Tat was slightly diminished. However, CycT1-V107E strongly bound to AFF4. It also did not assemble onto P-TEFb RNA targets - TAR RNA and 7SK snRNA. Importantly, HA-CycT1-V107E efficiently inhibited HIV replication in human T cell lines and in CD4(+) primary cells. This mutant also inhibited Tat-independent basal transcription and rapidly enforced transcriptional repression of HIV. Overall, this study defines a role for the cyclin box N-terminal residues of CycT1 in mediating contacts of P-TEFb with its interacting partners Cdk9, Hexim1 and AFF4. It also provides a mechanistic explanation for the inhibitory function of a unique CycT1-V107E on HIV gene transcription and signifies the importance of a functional P-TEFb and SEC unit in mediating HIV transcription.

## Results

### Association of HA-Cyclin T1 mutants with Cdk9 and Hexim1 in cells

**Figure 1:**
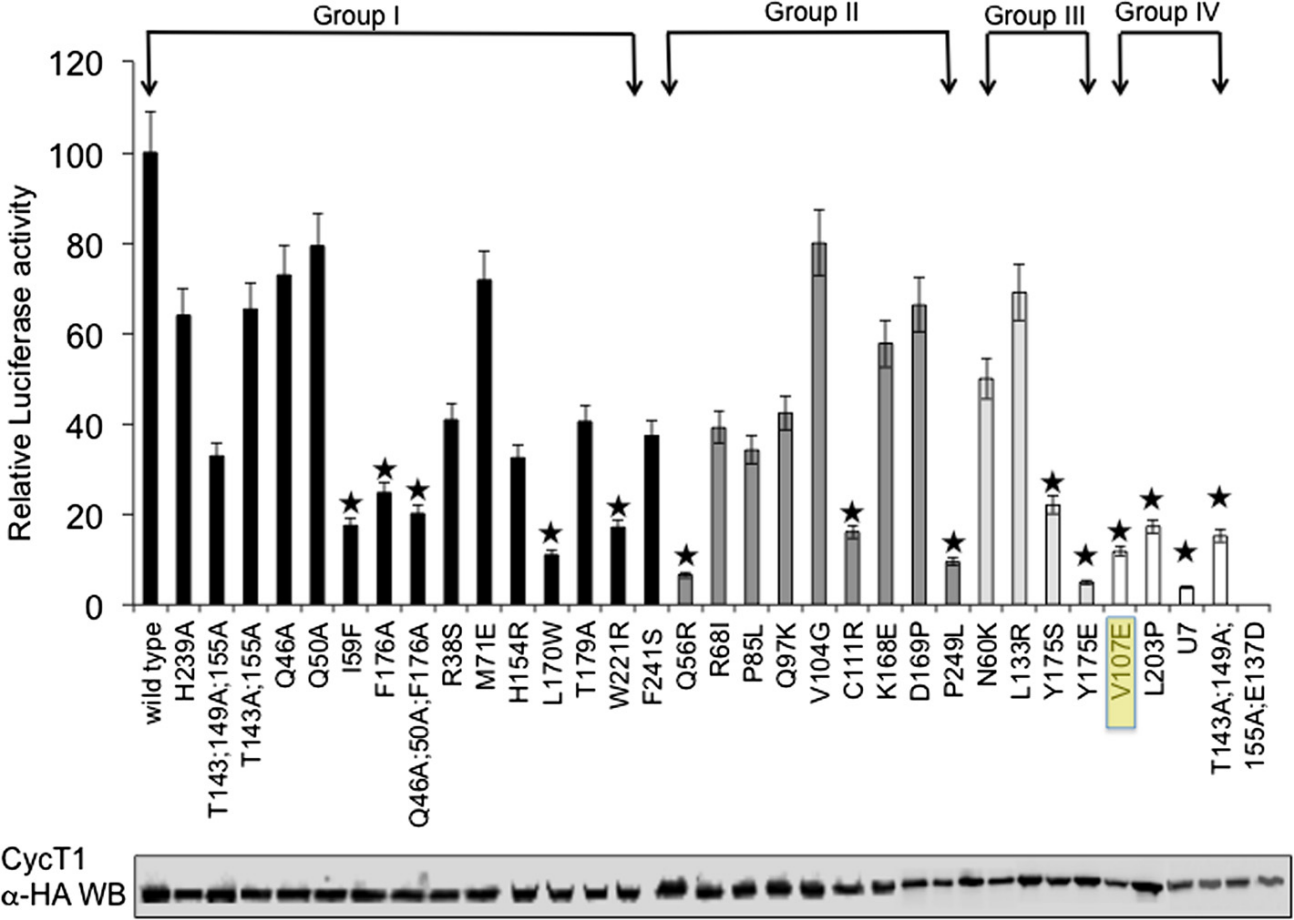
**Analysis of HIV Tat transactivation in murine cells upon expression of HA-CycT1 mutants.** 3T3 murine cells were co-transfected with the HIV-LTR-Luciferase reporter gene, HIV Tat and each of the HA-CycT1 mutants expressing plasmids. 48 hr. post transfection cells were harvested and their Tat-dependent luciferase activities were measured. Luciferase readings were normalized to *Renila* expression and data are presented relative to the readings obtained in cells that expressed human HA-CycT1-wild-type – set to 100 (assays were measured relative to cells that expressed HIV LTR-Luc reporter and Tat alone). HA-CycT1 mutants are divided to four groups according to their binding to Cdk9 and Hexim1 (Table 1). Asterisks mark specific HA-CycT1 mutants that were severely impaired in their ability to rescue Tat transactivation. Results are representative of the mean value of triplicate wells; error bars show ± SEM.

To identify residues in the cyclin box of cyclin T1 (CycT1) that are involved in association of CycT1 with Cdk9, Hexim1 or AFF4 of SEC transcription partners, as well as support HIV transcription, an error prone PCR-based mutagenesis was used to generate a pool of N-terminal human CycT1 mutants as described by Verstraete *et.al*. (submitted; Table 1). HA-tagged-CycT1 mutants were generated in the context of [1–280] residues, sub-cloned into a lentivector, and were transiently expressed in human HEK-293T cells. Western Blot (WB) analysis confirmed expression of HA-CycT1 mutants (Figure 1). Immuno-Precipitation (IP) experiments were then performed in cells to examine the association of HA-CycT1 mutants with host Cdk9, Hexim1 and SEC/AFF4. Binding efficiencies were compared to those of HA-CycT1-wild type (Table 1). Based on their abilities to associate with Cdk9 and Hexim1, HA-CycT1 mutants were divided into four groups (Table 1). Group I, consisted of HA-CycT1 mutants that bound to Cdk9 and Hexim1, as efficiently as wild-type Ha-CycT1. Group II mutants, exhibited weak binding to both Hexim1 and Cdk9. These include Q56R; R68I; P85L; Q97K; V104G; C111R; K168E; D169P, P249L HA-CycT1 mutants. Group III, consisted of HA-CycT1 mutants that did not bind to Hexim1, yet they bound to Cdk9. In some cases this interaction was weaker than that detected for HA-CycT1-wild type. This group included N60K, L133R, Y175E and Y175S. Importantly, these HA-CycT1 mutants were the first single-point mutations that have been reported to bind to Cdk9 but not to Hexim1. A last and interesting group, Group IV of HA-CycT1 mutants, lost their ability to associate with both Cdk9 and Hexim1. This group included HA-CycT1-U7 [28], V107E; L203P; and the quadruple mutant-[T143A;149A;155A;E137D]. All HA-CycT1 mutants in this group were impaired in their binding to AFF4/SEC, except HA-CycT1-V107E (Table 1). Overall, most N-terminal helices HA-CycT1 mutants bound to AFF4. Those that were impaired in their AFF4 binding, also did not bind Cdk9 or Hexim1 (or bound very weak to both), and as such were part of groups II-IV (N60K; R68I; L203P U7, [T143A;149A;155A;E137D]) [28].

**Table 1:** Summary of HA-Cyclin T1 point mutations

**Cyclin T1 mutation**	**Hexim1**	**Cdk9**	**AFF4**	**Tat activity [murine cells**^**1**^**]**	**LTR basal [human cells**^**2**^**]**	**Inhibition of Tat activity [human cells**^**3**^**]**
***Group I***						
wild type	+++	+++	+++	100	100	-
H239A	+++	+++	+++	64	110	-
T143;149A;155A	+++	+++	+++	33	108	-
T143A;155A	+++	+++	+++	65	107	-
Q46A	+++	+++	+++	73	103	-
Q50A	+++	+++	+++	79	111	-
159 F	+++	+++	+++	17	97	-
F176A	+++	+++	+++	25	54	-
Q46A;50A;F176A	+++	+++	+++	20	81	-
R38S	+++	+++	+++	41	69	-
M71E	+++	+++	+++	72	100	-
H154R	+++	+++	+++	32	123	-
L170W	+++	+++	+++	11	95	-
T179A	+++	+++	+	41	74	-
W221R	+++	+++	+	17	101	-
F241S	+++	+++	+++	37	115	-
***Group II***						
Q56R	+	+	+	7	104	-
R68I	+	+	-	39	105	-
P85L	+	+	+++	34	76	-
Q97K	+	+	+++	42	85	-
V104G	+	+	+	80	160	-
C111R	+++	+	+++	16	90	-
K168E	+	+	+++	58	125	-
D169P	+	+	+++	66	89	-
P249L	+	+	+++	9	109	-
***Group III***						
N60K	-	+	-	50	114	+
L133R	-	+	+++	69	70	-
Y175S	-	+++	+++	22	107	-
Y175E	-	+++	+++	5	69	-
***Group IV***						
V107E	-	-	+++	12	28	+
L203P	-	-	-	17	102	-
U7	-	-	-	4	141	+
T143A;149A;155A;E137D	-	-	-	15	92	-

HA-Cyclin T1 mutations were generated by random mutagenesis as described (Verstraete *et.al*.). Protein interactions between HA-CycT1 mutants and P-TEFb transcription partners (Hexim1; Cdk9; AFF4) were monitored in human HEK-293T cells, following over expression of the HA-CycT1 mutants. Cells were transfected with HA-CycT1 mutants and 48 hr. later were lysed and subjected to Immuno-Precipitation (IP) with α-HA antibody. Precipitated HA-CycT1 proteins were then analyzed by SDS-PAGE and WB for association with endogenous partners of P-TEFb - Hexim1; Cdk9 and AFF4. Binding efficiencies are presented as (+++) for efficient binding, which is comparable to HA-CycT1-wild type, (+) for weak binding, or (−) for lack of protein interactions respectively.

**Figure 2:**
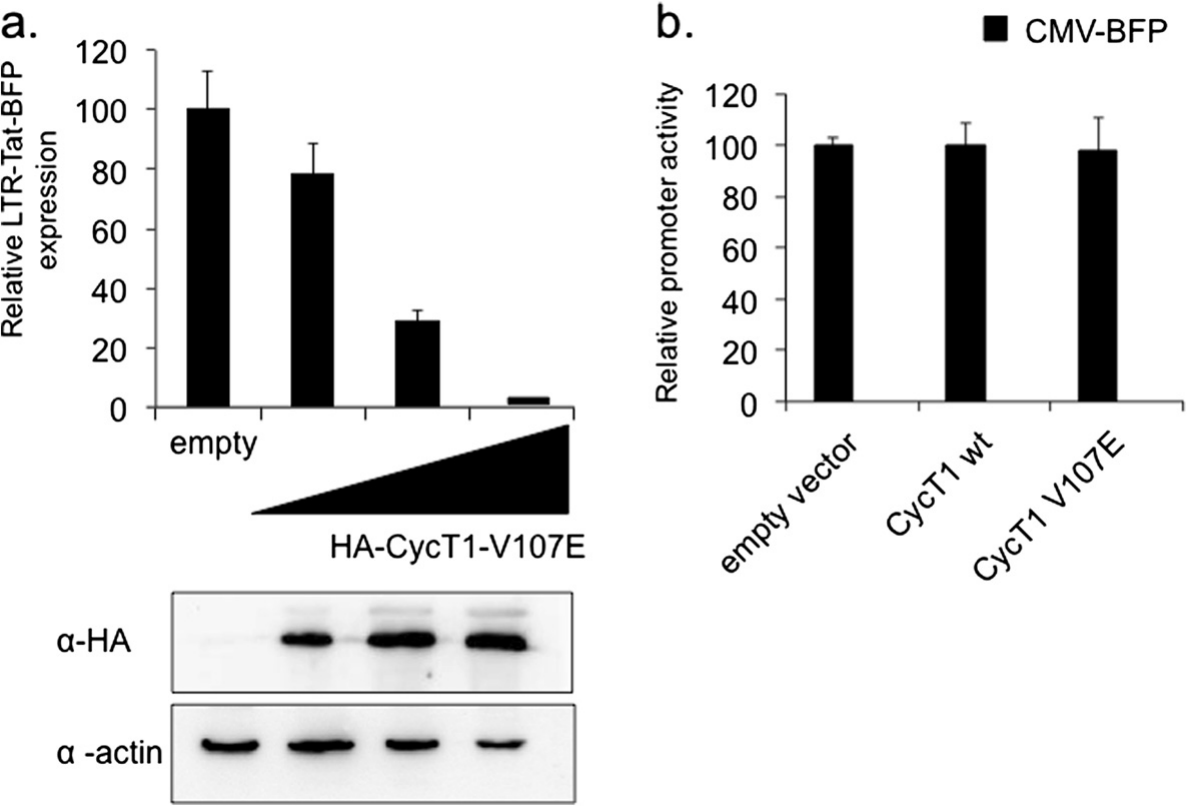
**HA-CycT1-V107E mutant inhibits HIV transcription in human cells. a)***HA-CycT1-V107E inhibits HIV-Tat dependent transcription from the HIV LTR promoter* - HEK-293T cells were transfected with increasing concentrations of the HA-CycT1-V107E mutant and the LTR-Tat-BFP provirus lentivector using Lipofectamin 2000. 48 hr. post transfection cells were harvested and the percentage of cells that expressed BFP was measured by FACS. Data are presented relative to cells that did not express HA-CycT1-V107E - set to 100. Lower panel shows Western blot that confirmed HA-CycT1-V107E expression and equal expression levels of actin. **b)***HA-CycT1-V107E does not inhibit basal transcription from the CMV promoter* - HEK-293T were co-transfected with HA-CycT1-wild type or HA-CycT1-V107E mutant in the presence of the CMV-BFP reporter promoter. 72 hr. post transfection, cells were harvested and their BFP expression was monitored by FACS. The percentage of BFP expressing cells in the presence of HA-CycT1 wild type was set to 100. Results are the average of there independent experiments. Error bars show ± SEM values.

(1) *Rescue of HIV Tat transactivation in murine cells* - Table 1 also summarizes the ability of HA-CycT1 mutants to activate Tat-dependent transcription from the HIV-LTR promoter in murine cells and corresponds to Data in Figure 1. Experiments were performed in 3T3 cells that were co-transfected with the HIV-LTR promoter, which drives a luciferase reporter gene, HIV Tat and HA-CycT1. Luciferase readings were normalized to *Renila* expression. Readings are presented relative to those obtained in cells that expressed HA-CycT1-wild-type - set to 100 (CycT1-wild type was set relatively to HIV-LTR-luciferase and Tat alone). Data is a representative of the mean value of triplicate wells; error bars show ± SEM.Ability of HA-CycT1 mutants to inhibit Tat dependent HIV transcription in human cells was also tested as presented in Figure 2.

(2) *Inhibition of Tat-independent HIV transcription in human cells by HA-CycT1 mutants* - cells were co-transfected with HIV-LTR-luciferase reporter gene and HA-CycT1 mutant 48 hr. post transfection cells were harvested and their luciferase activities were measured. Luciferase readings were normalized to *Renila* expression and are presented relative to the readings obtained in cells that expressed HA-CycT1-wild-type alone - set to 100. Results are representative of the mean value of triplicate wells; error bars show ± SEM**.**

(3) *Inhibition of Tat-dependent HIV transcription in human cells by HA-CycT1 mutants* - HEK-293T cells were transfected with two concentrations of HA-CycT1 mutants and the LTR-Tat-BFP reporter provirus 48 hr. post transfection, cells were harvested and the percentage of cells that expressed BFP was measured by FACS. Cells were also introduced with a pCDNA-CMV-GFP expression plasmid to control for transfection efficiencies by measuring percentage of GFP expressing cells via FACS. Symbols of inhibition (+); or lack of inhibition of Tat transactivation (−). Data are presented relatively to the effect of human HA-CycT1-wild type.

### Ability of HA-CycT1 mutants to support Tat transactivation in murine cells

To screen HA-CycT1 mutants for their ability to support Tat transactivation, experiments were performed in murine 3T3 cells (Figure 1). Murine CycT1 binds weakly to HIV Tat, thus cannot enhance Tat transcriptional activity. Ectopic expression of human CycT1 in these cells rescues Tat activity and stimulates HIV transcription [8]. HA-CycT1 mutants were transiently expressed in murine cells in the presence of the HIV-LTR-Luciferase (Luc) and HIV Tat. The ability of each of the HA-CycT1 mutants to enhance Tat transactivation was compared to that of wild type HA-CycT1 - set to 100. Expression of HA-CycT1 mutants was confirmed by anti-HA western blot in 3T3 cells. All HA-CycT1 mutants were impaired, to some extent, for their ability to support Tat activity in murine cells (Table 1). HA-CycT1 mutants that did not bind to Cdk9 or Hexim1 (Group IV) exhibited the weakest Tat transactivation levels. HA-CycT1-V107E; L203P, the quadruple mutant T143A;149A;155A;E137D and HA-CycT1-U7 mutant were the least efficient [28]. HA-CycT1 mutants from Group III, which did not bind Hexim1, yet bound to Cdk9, also showed decreased ability to support Tat transactivation in murine cells. HA-CycT1 mutants Y175E and Y175S were particularly worth noticing. HA-CycT1 mutants that bound weakly to both Hexim1 and Cdk9 (Group II), all exhibited lower levels of supporting Tat function compared to HA-CycT1-wild type. However, levels of Tat transactivation were variable and relatively high when compared to mutants of group IV. While HA-CycT1-V104G; D169P; K168E mutants supported Tat function to levels of above 50% relatively to human HA-CycT1-wild-type, Q56R, R68I, P85L, Q97K, C111R, and P249L HA-CycT1 mutants exhibited low efficiencies of Tat transactivation in 3T3 cells (Table 1; Figure 1). Q56R, C111R and P249L HA-CycT1 mutants were severely impaired in their ability to activate Tat function. Finally, HA-CycT1 mutants that bound to Hexim1 and Cdk9 at levels similar to those of the wild type HA-CycT1 (Group I) also exhibited variable levels of Tat transactivation in murine cells. Interestingly, HA-CycT1 mutants I59F, F176A, L170W, W221R, and the triple mutant Q46A;Q50A;F176A exhibited low levels of Tat transactivation in murine cells. Overall, point mutations in the cyclin box of CycT1 lead to decreased capacity to support Tat-dependent activation of HIV LTR in murine cells (Figure 1).

### Ability of HA-CycT1 mutants to support Tat-independent and Tat-dependent transcription in human cells

We further analyzed the ability of HA-CycT1 mutants to mediate Tat-independent basal transcription from the HIV LTR in human HEK293T cells. These experiments were performed by transiently transfecting each of the HA-CycT1 mutants and the HIV-LTR- luciferase reporter, in the absence of HIV Tat. Effects of HA-CycT1 mutants on basal transcription were determined relatively to ability of wild type HA-CycT1 to activate transcription activity from the HIV LTR promoter – set to 100 (Table 1). Generally, without Tat, CycT1-wild type weakly activated the HIV promoter – up to only about 2 fold. Most HA-CycT1 mutants presented comparable levels of stimulation of basal transcription from the LTR to those detected for wild type HA-CycT1 (Table 1). Some HA-CycT1 mutants - T179A, L133R and Y175E exhibited slightly lower levels of HIV LTR-activation - around 70% relative to cells that expressed HA-CycT1-wild type. Other HA-CycT1 mutants - H154R, K168E, and U7 showed slightly higher basal transcription activation of the HIV LTR relative to the HA-CycT1-wild type. These results however are in the range of assay error and should not be distinguished from the wild type activity levels. Nevertheless, one mutant, HA-CycT1-V107E that did not bind to Hexim1 or Cdk9 stood out among all others and could not activate basal transcription of the HIV promoter. Its activation reached only to 28% relative to HA-CycT1-wild type. This mutant was further analyzed in more details - see below.

HA-CycT1 mutants were next analyzed in human cells for inhibition effects on Tat-dependent transcription from the viral LTR promoter (Table 1). HA-CycT1 mutants were transiently expressed in HEK-293T cells together with HIV-LTR-Tat-BFP (Blue Florescence Protein) reporter provirus. Cells were then analyzed by FACS for their ability to inhibit HIV-LTR-mediated BFP expression. Most of the HA-CycT1 mutants did not affect HIV transcription in human cells (Table 1). However, there were two notable exceptions that displayed strong inhibitory effects on Tat-dependent transactivation in human cells. HA-CycT1-U7 (which consists of four point mutation and a deletion [28]) and HA-CycT1-V107E (Figure 2a). Both mutants were impaired for their binding to Hexim1 and Cdk9. However, only HA-CycT1-V107E bound AFF4/SEC, while HA-CycT1-U7 did not (Table 1). Interestingly as noted above, HA-CycT1-V107E also displayed inhibitory effects on Tat-independent transactivation of the HIV LTR in the absence of Tat. Further analysis also verified that inhibition of basal transcription from HIV LTR by HA-CycT1-V107E was specific, as transcription from a CMV-BFP promoter was not inhibited by either HA-CycT1-wild type, or HA-CycT1-V107E (Figure 2b).

### Association of CycT1-V107E with P-TEFb partners and its RNA targets

**Figure 3:**
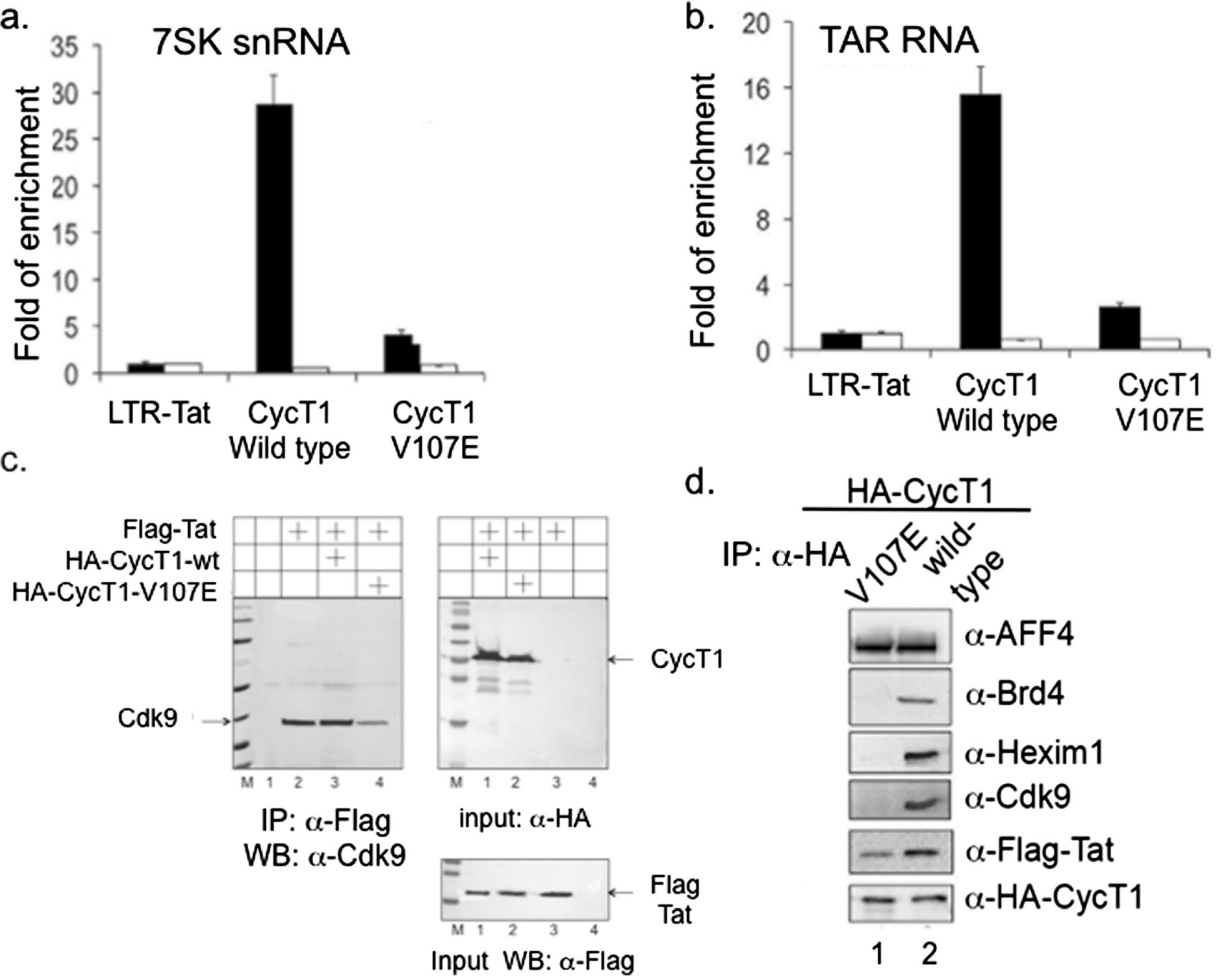
**Association of HA-CycT1-V107E with P-TEFb interacting partners and its RNA targets. (a + b)***HA-CycT1-V107E mutant does not bind TAR or 7SK snRNA in cells* – HEK-293T cells were co-transfected with HA-CycT1-V107E mutant, or HA-CycT1-wild type, HIV LTR-Luciferase and pCDNA-Myc-Tat plasmids. 48 hr. post transfection, cells were lysed and immuno-precipitated (IP) with either anti-HA antibody, or control non-immune anti-human IgG. RNA was extracted from IP and input samples (1%) and was then subjected to cDNA synthesis, which was further analyzed by real time PCR using 7SK-snRNA **(a)** and TAR specific primers **(b)**. Reactions were analyzed by real time PCR in triplicates and presented as fold of mean enrichment relatively to PCR results obtained for cells transfected with LTR-Luciferase alone - set to 1. Error bars show ± SEM values. **(c)***Association of HA-CycT1-V107E mutants with P-TEFb in cells* – HEK-293T cells stably expressing either HA-CycT1-wild type or HA-V107E-CycT1 were co-transfected with Flag-Tat using lipofectamin 2000 (Invitrogen). 48 hr. post transfection, cells were lysed and subjected to IP with α-Flag antibody. IP reactions were analyzed by WB with a Cdk9 antibody. α-HA WB represents 1% of input of HA-CycT1. **(d)***Association of HA-CycT1-V107E mutants with P-TEFb transcription partners in cells* – HEK-293T cells stably expressing either HA-CycT1-wild type or HA-V107E-CycT1 were lysed and subjected to IP with α-HA (left panel). IP reactions were analyzed by WB with the indicated antibodies.

Protein or RNA binding experiments in cells demonstrated that HA-CycT1-wild type bound efficiently to TAR RNA and to 7SK-snRNA (Figure 3a + b), as well as to Hexim1, AFF4, Brd4, Cdk9 and Tat (Figure 3d). In contrast, HA-CycT1-V107E neither associated with Hexim1 nor Cdk9 and Brd4 (Figure 3d), not with TAR and 7SK snRNA (Figure 3a + b). Its binding to Tat was slightly diminished, and it exhibited strong binding to AFF4/SEC. These results were in agreement with the Hexim1-binding deficiency of HA-CycT1-V107E (Figure 3d). To better characterize the mechanism of CycT1-V107E-mediated inhibition of HIV transcription, stable expression of HA-CycT1-wild type, or V107E was obtained in HEK-293T by lentiviral transduction and drug selection. The incorporation of Cdk9 onto the Tat-CycT1 complex was monitored in these cells, upon expression of Flag-Tat. Cells were subjected to anti-Flag IP, and the efficiency of Cdk9 associated with Tat and HA-CycT1-wild type was analyzed. As shown, expression of HA-CycT1-V107E and Tat, led to lower expression levels of Cdk9 in the complex, compared to cells that expressed HA-CycT1-wt. These results imply that the CycT1-V107E squelches Tat from the P-TEFb (Figure 3c).

### HA-CycT1-V107E mutant inhibits HIV replication in T cells and in primary CD4(+) T cells

**Figure 4:**
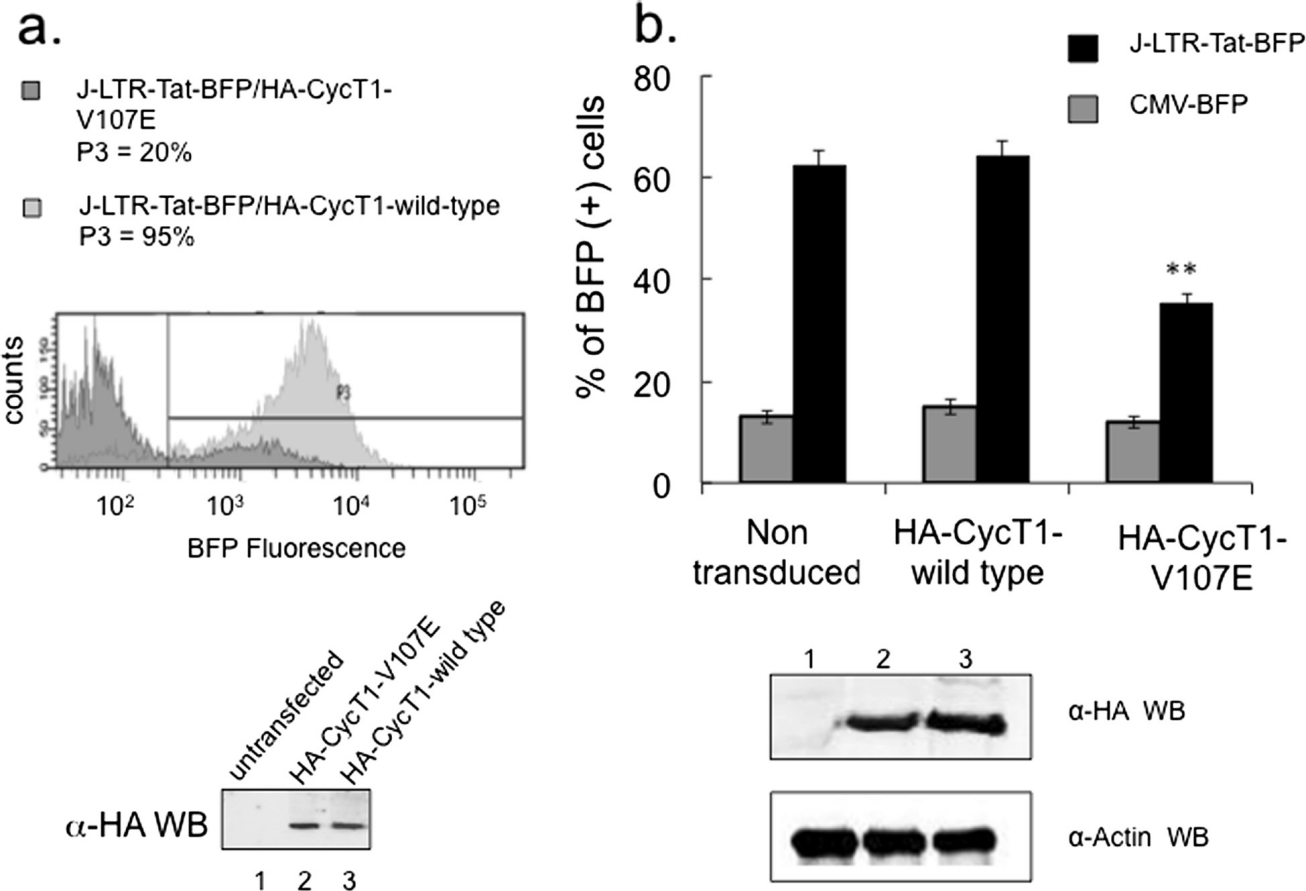
**HA-CycT1-V107E mutant inhibits HIV transcription in T cells and primary CD4(+) cells. a)***HA-CycT1-V107E inhibits HIV replication in human T cell line* - Jurkat T cells that harbor an integrated LTR-Tat-Rev-BFP provirus and express low levels of BFP (J-LTR-Tat-BFP), thus serve as model for viral latency, were transduced with lentiviruses expressing either HA-CycT1-wild type or HA-CycT1-V107E. Transduced cells stably expressing human CycT1 were generated following puromycin selection. J-LTR-Tat-BFP cells that did not express CycT1 were used as control. Cells were analyzed by FACS for their LTR-BFP expression. Presented is a FACS spectra dot-blot analysis for J-LTR-Tat-BFP cells expressing HA-CycT1-V107E (J-LTR-Tat/HA-CycT1-V107E; dark grey), or cells that expressed HA-CycT1-wild type (J-LTR-Tat/HA-CycT1 wild type; light grey spectra). The P3 gate was set based on J-LTR-Tat-BFP cells that did not express CycT1. Data is a representative of three separate experiments. Bottom panel shows HA-CycT1 protein expression levels in Jurkat cells as determined by anti-HA-WB. **b)***HA-CycT1-V107E inhibits HIV replication in primary CD4(+) T cells* - Human CD4(+) T primary cells were isolated from naïve PBMCs and stimulated with CD3/CD28 beads for 48 hr. Isolated cells were then transduced with either HA-CycT1-V107E or HA-CycT1-wild type expressing lentivirus. 48 hr. post transduction, cells were infected with HIV-LTR-BFP, or CMV-BFP lentivirus at MOI of 1. BFP-mediated viral replication was analyzed by FACS to monitor effects of HA-CycT1 expression. Expression of HA-CycT1-wild type or HA-CycT1-V107E was confirmed by anti-HA WB.

Inhibitory effects of HA-CycT1-V107E on HIV transcription were further investigated in human T cell lines (Figure 4). For this purpose, Jurkat (J)-LTR-Tat-BFP T cells, which harbor a transcriptionally silenced HIV-LTR-Tat-BFP integrated provirus, were utilized, similarly to J-LTR-Tat-d2EGP cells that were previously descried by the Karn lab [36]. The basal LTR expression in these cells was relatively low - close to 10% and thus, cells were suitable for studying viral latency. Jurkat (J)-LTR-Tat-BFP T cells that stably expressed either wild type or V107E HA-CycT1 were generated by lentiviral transduction (J-LTR-Tat-BFP/HA-CycT1-V107E, or J-LTR-Tat-BFP/HA-CycT1-wild type). 48 hr. post transduction, cells were subjected to selection with puromycin for additional 3-10 days to obtain HA-CycT1 stable cells. HA-CycT1 expression was validated by western blotting (Figure 4a-lower panel). Stable cells expressing HA-CycT1 (wild type or V107E) were then analyzed by FACS for their HIV-Tat-LTR-BFP expression. While close to 100% of LTR-Tat-BFP/HA-CycT1-wild-type cells expressed BFP, only 20% of J-LTR-Tat/HA-CycT1-V107E cells expressed BFP. We conclude that stable expression of HA-CycT1-V107E inhibits HIV transcription in J-LTR-Tat-BFP cells, while HA-CycT1-wild-type activates HIV transcription in these cells (Figure 4a; compare histogram for LTR-Tat-BFP/HA-CycT1 wild type - light grey; *versus* J-LTR-Tat-BFP/HA-CycT1-V107E -dark grey).The ability of HA-CycT1-V107E to inhibit HIV replication was further validated in human CD4+ primary T cells (Figure 4b). CD4(+) T cells were isolated and stimulated for 3 days with CD3/CD28 T cell expanders beads, supplemented with IL-2. Following activation, cells were transduced with lentiviruses expressing either HA-CycT1-V107E, or HA-CycT1-wild type and cells were propagated for additional 3 days. The expression of HA-CycT1-V107E, or HA-CycT1-wild type in CD4(+) primary cells was validated by western blotting, which confirmed efficient CycT1 gene marking (Figure 4b). Following, HA-CycT1 stable CD4+ T cells were challenged with VSV-G pseudotyped HIV-LTR-Tat-BFP lentivirus at MOI of one, and at 96 hr. post infection HIV replication was analyzed by FACS. As control, cells were transduced with a CMV-BFP lentivirus, which is not dependent on Tat for full promoter activation and generally displayed lower transcription activation. As shown, expression of the HA-CycT1-V107E mutant in CD4(+) primary cells led to a 60% inhibition of HIV replication, relative to cells that expressed HA-CycT1-wild type. Expression of either HA-CycT1 proteins did not affect the transcription of the CMV-BFP lentivirus (Figure 4b).

### HA-CycT1-V107E mutant enforces transcriptional repression of HIV in T cell lines

**Figure 5:**
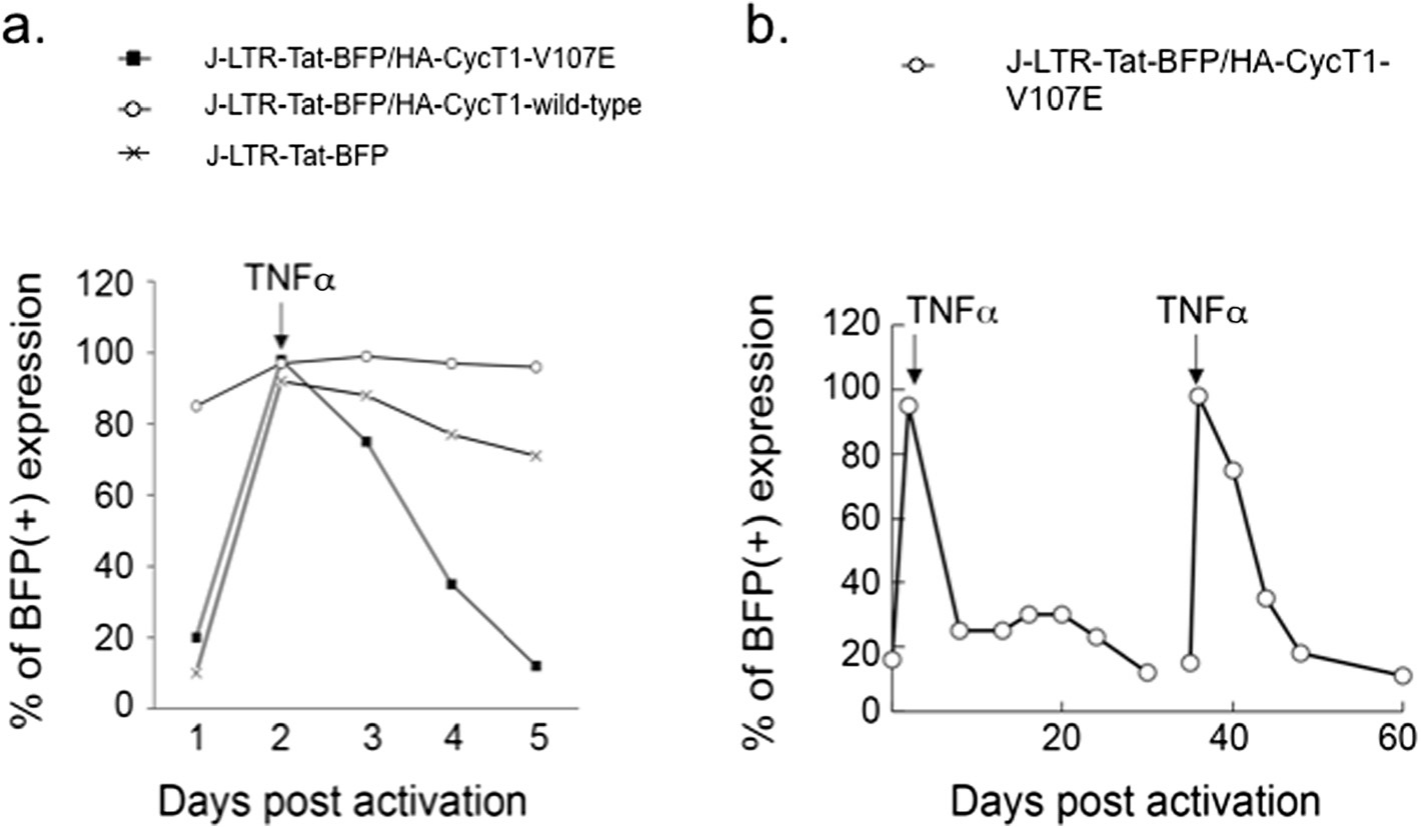
**HA-CycT1-V107E mutant enforces repression of HIV transcription. a)** J-LTR-Tat-BFP control cells or J-LTR-Tat-BFP cells that stably express either HA-CycT1-wild type, or HA-CycT1-V107E (J-LTR-Tat-BFP/HA-CycT1-wild type; J-LTR-Tat-BFP/HA-CycT1-V107E) were activated with TNFα 24 hr. post activation cells were washed and further maintained for additional two weeks. At the indicated time points post activation, cells were analyzed for their LTR-BFP expression by FACS. **b)** J-LTR-Tat-BFP cells that stably express HA-CycT1-V107E (J-LTR-Tat-BFP/HA-CycT1-V107E) were activated with TNFα and cells were maintained for additional growth. At the indicated time points post activation, BFP expression was monitored by FACS. At day 35-post initial activation, cells were again activated with TNFα. Cells were maintained for additional 25 days (60 days post initial activation), during which, HIV-LTR-BFP expression was measured by FACS.

The ability of HA-CycT1-V107E to enforce HIV transcriptional repression was also tested following activation of Jurkat cells with TNFα over longer periods of time (Figure 5). Upon TNFα activation, 100% of J-LTR-Tat-BFP stable cells (either HA-CycT1-wild type or HA-V107E) were activated and expressed BFP, implying that the HIV provirus in these cells was integrated, but transcriptionally silent. Control J-Tat-BFP cells were also activated with TNFα, and 100% of cells expressed BFP. Following TNFα activation, cells were further grown for additional 10–12 days and their LTR-Tat-BFP expression was monitored by FACS at the indicated time points post activation (Figure 5a). While cells that expressed HA-CycT1-V107E quickly reverted back to basal LTR transcription levels by day 12-post activation with TNFα, J-LTR-Tat-BFP that expressed HA-CycT1-wild type remained transcriptionally active and 100% of cells expressed BFP. In fact most of J-LTR-Tat-BFP/HA-CycT1-wild type cells died at day 12-post infection (Figure 5a). TNFα activated control cells that did not express CycT1, gradually lost their BFP expression in the 12 days time frame, but still remained highly active. These results demonstrate that HA-CycT1-V107E mutant efficiently represses HIV transcription from the LTR and enforces viral transcription silencing (Figure 5a).To evaluate effects of HA-CycT1-V107E on HIV transcription along longer periods of time points, J-LTR-Tat/HA-CycT1-V107E stable cells were treated with TNFα, which led to activation of HIV transcription and reversion to basal levels by day 12 post activation. J-LTR-Tat-BFP/HA-CycT1-V107E stable cells were kept growing and at day 35 post initial activation were activated again with TNFα. HIV transcription was further monitored by FACS - up to 60 days post-initial activation (Figure 5b). Following the second TNFα activation at day 35, HA-CycT1-V107E stable cells were activated but quickly reverted back to basal HIV transcriptional levels and remained at these low levels at 60 days post infection (Figure 5b).

## Discussion

In the current study a structural-biochemical analysis was performed to determine the significant role of the CycT1 cyclin box domain in mediating binding of P-TEFb to its transcription partners - Hexim1, SEC/AFF4, Cdk9 and HIV Tat. CycT1 mutants were generated via random PCR, and their abilities to support Tat transactivation in murine cells and to mediate Tat independent (basal) and Tat dependent transcription from the HIV LTR promoter in human cells were analyzed. This analysis led to the identification of a unique mutation - a conserved Valine (V) at position 107 - which was replaced with Glutamate (E). CycT1-V107E was impaired for binding to Hexim1, Cdk9 and Brd4, and its binding to HIV Tat was slightly diminished. It also did not bind to TAR RNA, or 7SK-snRNA. Consequently, CycT1-V107E could not support Tat transactivation in murine cells and efficiently inhibited basal Tat-independent and Tat-dependent HIV transcription from the HIV LTR promoter in human cells. Inhibition of HIV transcription by HA-CycT1-V107E mutant was specific as it did not the inhibit CMV promoter.

**Figure 6:**
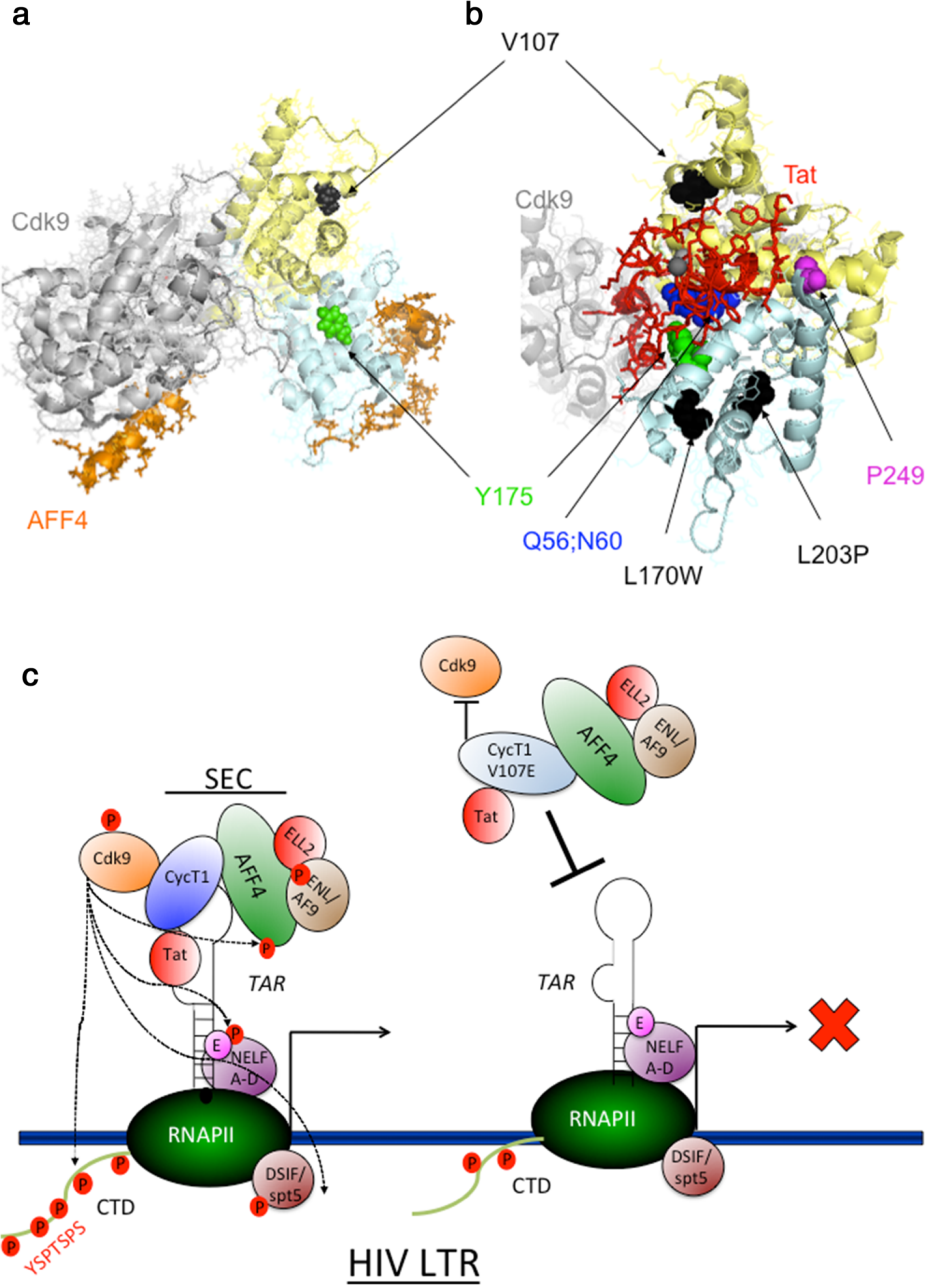
**Illustration of CycT1-V107E on the 3D structures of P-TEFb. a)** The V107E mutation in CycT1 was positioned on the structure of P-TEFb and AFF4/SEC. It is located on the H4 helix of the cyclin box (pale yellow) and in distance from the AFF4 binding surface and the binding region of CycT1 to Cdk9 (grey) [18]. **b)** Cyclin T1 mutations were positioned on the N-terminal helices in the cyclin box of the 3D structure of Tat and P-TEFb (Cdk9/CycT1) [18,30]. The N-terminal CycT1 cyclin fold is in pale yellow, the C-terminal one is in pale cyan, Tat is in red and Cdk9 is in light grey. This structure shows that Tat adopts a structure and the T-loop of the Cdk9. Mutations that abolished Tat transactivation in murine cells are also presented. The V107 residue is indicated in black. **c)***Model of CycT1-V107E mode of function* - CycT1-V107E does not bind Hexim1 and Cdk9, as well as to TAR RNA and 7SK snRNA. However, its binding to Tat and AFF4 is not impaired. As a result, both Tat and SEC are squelched from the HIV promoter, leading to repression of both Tat-independent and Tat-dependent HIV transcription. Significantly, CycT1-V107E allows the dissection of binding surfaces that are involved in either Tat or Hexim1 binding. Moreover, it signifies the importance of SEC for HIV transcription and can serve as a tool to dissect effects of P-TEFb and SEC on either Tat independent and Tat dependent transcription.

Inspection of the recently published P-TEFb-Tat-TAR three-dimensional structures illustrates that the Valine at position 107 does not contact with TAR and is not part of the TRM (Tat-TAR Recognition Motif) on CycT1. CycT1-V107 also does not reside within the Hexim1/Tat-CycT1 binding surface. However, it is located adjacent to the hydrophobic pocket on CycT1, which is formed by the two N-terminal helices of the cyclin box and constitutes the binding surface of CycT1 to Tat and Hexim1 [18,29,30,34,35]. Indeed, positioning the V107E mutation on the 3D structure of the CycT1-Tat fusion protein from EIAV confirms that it is located on the H4 helix at the first cyclin box. Residues on helices H4 and H5 and the Tat-interacting loop define the Hexim1/Tat hydrophobic binding pocket. Together with TRM at the C-terminal helix of CycT1-cyclin box, this pocket forms the binding site for Tat [35]. As such, V107 may well interact with Tat, as it is in close proximity to I105 and A108 residues that are part of the Tat-interacting loop. In addition, the last visible residue (Leu 68) of EIAV-Tat in this model is also inserted into the defined hydrophobic pocket behind helix H4 that carries V107. The MRAIL motif - YRQQ_40_A in the N-terminus cyclin box repeat of CycT1 (which on Cdk2 is located on helix H1 and is known to be part of the recognition motif of the cyclin to its substrate or inhibitors), also constitutes part of the Tat binding surface of CycT1 [37,38]. Overall, the change from a hydrophobic-non-charged Valine residue to a negatively charged and hydrophilic Glutamate residue may disrupt the conformation of the Tat/Hexim1 pocket and thus, explain impaired binding of CycT1 to Hexim1, Tat and possibly TAR [35]. The crystal structure of the Tat-P-TEFb complex, containing HIV-1 Tat, Cdk9 and human CycT1 also demonstrates that Tat adopts a structure complementary to the surface of P-TEFb and makes extensive contacts, mainly with the CycT1, but also with the T-loop of the Cdk9 subunit (Figure 6b). We think that the fact that CycT1-V107E does not bind Cdk9, also may explain its reduced binding of CycT1 to Tat [30]. Finally, the structure of the tripartite complex consisting of the recognition regions of Cdk9, CycT1 and AFF4 also shows that AFF4 binds the C-terminal domain of the cyclin box of CycT1, at the opposite surface of the Cdk9 binding region and far away from helix H4 that carries V107. This explains the ability of the V107E CycT1 mutant to strongly bind AFF4 (Figure 6a) [18]. The recent published structure of HIV Tat complexed with P-TEFb and AFF4 confirms the above data and clearly shows that upon formation of the Tat-AFF4 and P-TEFb complex, concerted structural changes in AFF4 occur *via* a shift of helix H5' of CycT1 and the α-3_10_ helix of AFF4. This structure shows that the TRM of CycT1 interacts with both Tat and AFF4, leading to the exposure of arginine side chains for binding to TAR RNA. Furthermore, modeling of Tat Lys28 acetylation suggests that the acetyl group would be in a favorable position for H-bond formation with Asn257 of TRM, thereby stabilizing the TRM in CycT1, and provides a structural basis for the modulation of TAR RNA binding by acetylation of Tat Lys28 [39].

Our analysis demonstrated that other mutants within the Tat-Hexim1 “binding pocket” were also impaired for Tat transactivation in murine cells. CycT1-Q56, I59F, the triple [Q46A;Q50A;F176A], L170W, W221R, C111R and P249 mutants are all positioned on the opposite helix of CycT1, around the Y175 residue and according to our analysis could not support Tat function in murine cells. CycT1-Y175 is positioned within the groove that is located between the two cyclin folds of CycT1 and forms an H-bond with Tat. As such, it defines the surface opposite of the Tat binding surface. Q46 and Q50 belong to the Y175 H-bond network and were previously reported to disrupt Tat binding and transactivation [40]. Importantly, the Y175-HA-CycT1 mutants, as well as other members of Group III are the first single-amino-acid mutations to be reported as impaired for binding of CycT1 to Hexim1 and Tat (Verstraete *et.al* under revision). HA-CycT1-Y175S or Y175E were also impaired for rescue of Tat transactivation in murine cells and binding to RNA (Verstraete *et.al* under revision). However, other HA-CycT1 mutants in Group III, N60K and L133R, did not bind to Hexim1, but supported (to some extent) Tat function, despite weak binding to Cdk9. These results imply that the Hexim1/Tat binding surface on CycT1 is not completely overlapping. Overall, our results signify the important role of the N-terminal helices of CycT1 in the formation of the Tat/Hexim1 binding pocket. Mutations in both helices disrupt the pocket and inhibit Tat/Hexim1 and also Cdk9 binding, thus inhibit Tat function.

The current study also shows that most HA-CycT1-N-terminal mutants bound AFF4/SEC. Thus, N-terminal helices of CycT1 may not contribute much to the binding of CycT1 to AFF4/SEC. Those CycT1 N-terminal mutants that did bind AFF4, also could not associate with Cdk9 or Hexim1 (Group IV). This implies that Cdk9 and/or Hexim1 plays a role in the association of P-TEFb with SEC, and assembly of these complexes on the HIV promoter. Indeed structural studies confirm show that the Hexim1/Tat binding surface on CycT1 does not overlap with that of AFF4 [18]. Moreover, these studies also demonstrate that CycT1-N-terminus 300 residues are mapped for AFF4 binding [18]. Our study significantly identifies the N-terminal residues of CycT1 that are involved in AFF4/SEC recruitment *versus* those that are contributing to Tat-Hexim1 binding. Importantly, HA-CycT1 mutants that exhibited impaired binding to AFF4 were part of Group IV that did not associate with Cdk9 or Hexim1 in cells.Effects of HA-CycT1-V107E on HIV transcription were also tested in human Jurkat T cells that stably express integrated HIV-LTR-Tat BFP (J-LTR-Tat-BFP). These cells express low levels of BFP, thus serve as model cell lines for HIV latency. Stable expression of HA-CycT1-V107E in J-LTR-Tat-BFP cells (J-LTR-Tat-BFP/CycT1-V107E) inhibited HIV transcription and enforced entrance of HIV into latency. In contrast, expression of HA-CycT1-wild-type in these cells led to enhancement of HIV transcription (Figure 5). As expected, activation of cells with TNFα led to an overall enhancement of HIV transcription in all cells that expressed either wild type or V107E HA-CycT1. However, while expression of CycT1-V107E accelerated transcription silencing of HIV in a 12-day post activation time-frame, HIV transcription in cells that expressed wild type-HA-CycT1 remained activated and finally went through apoptosis (Figure 5). Similarly, HIV transcription in J-LTR-Tat-BFP control cells that did not express HA-CycT1 gradually decreased, but still remained high in the 12-day time frame post activation (Figure 5). We conclude that CycT1-V107E accelerates silencing of HIV transcription and enforces latency. Repression of HIV transcription in J-LTR-Tat/HA-CycT1-V107E cells was also detected in longer time points. A second activation of J-LTR-Tat-BFP/HA-CycT1-V107E cells, resulted in full restoration of HIV transcription. However, as for the first cycle of TNFα activation, HA-CycT1-V107E expressing cells quickly reverted back to a resting state and remained as such up to 60 days post initial activation with TNFα (Figure 5). Inhibitory effects of CycT1-V107E mutant were also tested in human CD4(+) primary cells. In these cells, stable expression of CycT1-V107E mutant also repressed HIV replication - up to 60% (Figure 4b). Current experiments are attempting to improve gene marking of CD4(+) primary cells and elevate the repressive effects on transcription of CycT1-V107E (Figure 4b).This study aims to understand the mode of function of CycT1-V107E. We hypothesize that strong binding of CycT1-V107E to AFF4/SEC and slightly weaker binding to Tat, but not to RNA, Hexim1 and Cdk9, may squelch SEC and Tat from the viral promoter, prompting efficient silencing of HIV transcription in human cells (Figure 6c). These results signify the role of SEC, P-TEFb and Tat in regulating HIV transcription and the importance of SEC and Tat in promoting HIV latency. The inability of HA-CycT1-V107E mutant to assemble on Hexim1 and 7SK snRNA may also shift the equilibrium of P-TEFb towards its inactive complex, enhancing the inhibition of P-TEFb activity and repression of transcription from the HIV promoter.

Binding experiments of HA-CycT1 to Hexim1, Cdk9 Tat, TAR and 7SK snRNA, identified other mutants that belong to the same group of CycT1-V107E (Group IV). These mutants did not bind Cdk9 and Hexim1 and did not support Tat transactivation in murine cells. These include HA-CycT1-L203P, U7 and the quadruple mutant T143A;T149A;T155A;E137D. Interestingly, HA-CycT1-V107E was the only mutant in this group that effectively bound to AFF4 subunit of SEC and yet exhibited dominant negative inhibition phenotype on HIV transcription in human cells. These results suggest that in the synergistic transcriptional effects of P-TEFb and SEC on the activity of RNA polymerase, the recruitment of SEC to the HIV promoter is highly significant for enhancing viral transcription.

Overall, this work defines the binding surfaces of CycT1 to Hexim1 which may be spreadable from those of Tat. It also describes for the first time a V107E-CycT1 mutation that effectively inhibits Tat independent and Tat dependent transcription from the HIV promoter. As CycT1-V107E does not bind Cdk9 and TAR, but strongly binds to AFF4 and its association with Tat is slightly impaired, it can serve as a unique tool to dissect the effects of SEC on HIV transcription.

## Conclusions

This work defines N-terminus binding residues of CycT1 to Cdk9, AFF4, RNA and Hexim1. Binding of Hexim1 to CycT1 could be dissected from that of Tat. Significantly, our analysis also describes for the first time a unique V107E-CycT1 mutation that effectively suppresses Tat independent and Tat dependent transcription from the HIV promoter in both human T cell lines and in CD4(+) primary T cells. CycT1-V107E does not bind Hexim1, Brd4, and Cdk9, and does not assemble on TAR RNA, or 7SK snRNA. However, it binds AFF4 of SEC and associates with Tat. As such, it serves as a unique tool for studying the effects of SEC on HIV transcription that are P-TEFb independent, and signifies the importance of a functional P-TEFb and SEC in supporting HIV transcription.

## Methods

### Plasmids

HA-CycT1 mutants were generated in the context of the CycT1-(1–280) residues, and cloned in a pHAGE-CMV-IRES-puromycin lentiviral vector (a generous gift of the Mulligan lab - HMS). The HA-tag was positioned at the C-terminus of the protein. Stable cells expressing CycT1-V107E were generated following transduction, by puromycin selection. pCDNA3-myc-Tat and HIV-LTR-Luciferase were used for transient transfection and analysis of CycT1 effects in murine cells. pRL-*Renila* was used in these assays for control of transfection efficiencies (Promega). In human cells, LTR-Tat-BFP reporter provirus was used (based on the pHRP-PNL-d2EGFP a generous gift of J. Karn; the BFP protein replaced the deEGFP) [36]. pCDNA3-CMV-GFP expression plasmid was used for transfection efficiencies in these assays. For protein and RNA binding experiments between HA-CycT1 and P-TEFb partners, HEK293T cells were co-transfected with the pHAGE-CMV-HA-CycT1 lentiviral vector and pCDNA3-Flag-Tat. For infection of Jurkat cells and human primary CD4(+) cells, VSV-G pseudotyped lentivirus expressing a Blue Florescence Protein (BFP) reporter under the control of the HIV promoter was used (HIV-LTR-BFP).

### Cells

HEK-293T human cells; 3T3 murine cells and Jurkat T cells were respectively maintained in complete 10% FCS DMEM or RPMI-1640, at 37°C with 5% CO2.

### Cyclin T1 mutants

A collection of CycT1 mutants cloned in yeast hybrid vector were generated by Verstraete *et. al.* (submitted). Mutant CycT1-cyclin box domain (1–280 residues) were amplified by 30 cycles of PCR. The reverse 3’ primer also contained HA-tag sequence for the expression of a C-terminus HA tag. PCR fragments were digested with BamHI/NotI and inserted into the same sites in pHAGE lentiviral vector.

#### Production of HIV lentiviruses

VSV-G pseudotyped single round particles were produced by CaPO_4_ co-transfection into HEK-293T of the lentiviral transvector (either the proviral LTR-Tat-BFP, or HA-CycT1 mutants) plasmid, HIV gag/pol, pRev, pTat and the VSV-G envelope. 48 hr. post transfection, cell supernatant was harvested, centrifuged and filtered through 0.45 μ filter. Lentiviruses were concentrated by ultracentrifugation for 2 hr. at 25 K rpm, and the pellet was suspended in PBS. Aliquots of viral stocks were frozen. Titer of viruses was determined by transduction of HeLa cells with viral serial dilutions. 48 hr. post transduction, cells were harvested and analyzed by FACS for BFP expression.

#### Binding of CycT1 mutants in cells

HEK-293T cells grown in 10 cm plate were transfected with 10 μg of the indicated pHAGE-HA-CycT1 plasmid, using Lipofectamine 2000 (Invitrogen). Cells were also transfected with 5 μg of LTR-Luc, 5 μg FLAG-Tat and 2 μg of pCDNA-CMV-GFP to measure transfection efficiencies (48 hr. post transfection, 90% of cells were green). 48 hr. post transfection, cells were lysed with an hypotonic buffer (0.1% Triton; 20 mM Tris-Cl pH 7.6; 200 mM NaCl; 0.72 mM EDTA; 10% Glycerol; 14 mM β-Mercaptoethanol (added fresh before use, 1:1000); protease inhibitors (PI; Sigma; 1/200 dilution). Lysates were incubated on ice for 1 hr. and then and centrifuged at 14,000 rpm for 10 min at 4°C. Cleared supernatants were then incubated overnight with gentle rocking with 1 μg of α-HA antibody (Abcam ab-9110). Following, lysates were incubated with BSA-pre-blocked protein A-sepharose beads (Invitrogen) at 4°C for 2 hr. with gentle rocking. Beads were extensively washed ×4 times with washing buffer (lysis buffer containing 0.05% Triton), and were then precipitated by centrifugation at 3000 rpm at 4°C. Immuno-Precipitated (IP) proteins were separated on SDS-PAGE and analyzed by western blotting (WB) with the indicated antibodies diluted in PBS; α-Hexim1 (ab25388); α-Cdk9 (ab6544); α-AFF4 (ab 57077,); α-Flag (M2-Sigma); α-Brd4 (ab75898); Secondary antibodies were α-mouse (Jackson-115035062) and α-rabbit (Jackson-111035045).

### Analysis of Tat transactivation in murine and human cells

To measure the ability of HA-CycT1 mutants to support HIV Tat transactivation in murine cells, the following DNA plasmids were transiently co-transfected into 3T3 cells, using Lipofectamine 2000 (Invitrogen). LTR-Luc (0.1 μg); pCDNA-myc-Tat (0.2 μg); pHAGE-HA-CycT1 mutant, or pHAGE-HA-CycT1 wild type (2 μg). 6 hr. post transfection, media was replaced with fresh complete DMEM. Cells were harvested 48 hr. post transfection and lysed with 100 μl of luciferase lysis buffer (Promega). 20 μl of sample was used for luciferase measurements according to the manufacturer instructions. Activation of HIV-Tat activity upon expression of human CycT1 was used as control - set to 100. All luciferase activities were measured relatively to the wild type CycT1. Transfection efficiencies were normalized between wells by monitoring *Renila* expression following transfection of pRL *Renilla* Luciferase Reporter Vector (200 ng/24 well).

#### In human cells

Tat-independent inhibitory effects of HA-CycT1 mutants were monitored by transiently co-transfecting pHAGE-HA-CycT1 mutant (2 μg) and the HIV-LTR-luciferase reporter (0.1 μg). pRL *Renilla* Luciferase Reporter Vector (Promega) (200 ng/24well) was used to measure transfection efficiencies and luciferase readings were normalized to the *Renilla* expression.

For analysis of inhibition of Tat-dependent transactivation pHAGE-HA-CycT1 mutants were transfected (2-4 μg) with the LTR-Tat-BFP lentiviral vector (1 μg). pCDNA3-CMV-GFP (0.1 μg) was also transfected into cells, to monitor transfection efficiencies 48 hr. post transfection, cells were harvested and their BFP expression were measured by FACS relatively to cells that expressed wild type HA-CycT1. Data were presented as (−) or (+) representing inhibitory affects on HIV transcription in human cells.

For effects of HA-CycT1 mutants on the NF-kB or CMV promoters, HA-CycT1 mutants were transfected with into human HEK-293T cells (2-4 μg) together with either the NF-kB-Luciferase promoter or the CMV-BFP promoter (1 μg). Cells were harvested 48 hr. post transfection and analyzed for their luciferase activity or BFP expression by FACS. Data are presented relatively to effects of CycT1-wild type –set to 100.

### Generation of Jurkat T cell line models for HIV latency that express HA-CycT1 and analysis of effects on HIV transcription

Jurkat (J)-LTR-Tat-BFP cells were generated following transduction of VSV-G pseudotyped HIV-LTR-Tat-Rev-BFP lentivirus. In these lentiviruses, Tat and Rev are expressed in cis under the control of the HIV LTR promoter, as described by the Karn lab [36]. The BFP reporter gene replaced the 2dEGFP gene which was inserted downstream of the Tat-Rev genes. Transduced cells were maintained for growth for a long period of time (up to a month), allowing the integrated HIV provirus to enter transcription silencing. In these cells, basal HIV transcription reached to 10%, and upon activation with TNFα, 100% of cells expressed BFP - implying transcription silencing of the integrated Tat-BFP provirus. For stable expression of HA-CycT1 in J-LTR-Tat-BFP, cells were transduced with pseudotyped lentiviruses expressing CycT1 (wild type or V107E) and 48 hr. post transduction, cells were selected on puromycin for a week. HA-CycT1 expression was validated by Western blot and effects on HIV transcription were monitored by FACS, measuring expression of BFP.

To analyze effects of HA-CycT1-V107E on HIV transcription following TNFα activation and over longer time frames, J-LTR-Tat-BFP/CycT1-wild type, or CycT1-V107E mutant were activated with 10 μg/ml TNFα for 24 hr. BFP expression was monitored by FACS at the indicated time points. For longer-time points, cells were again activated again by TNFα for 24 hr. at 30 days post initial activation and cells were further analyzed for their BFP expression for additional 30 days (60 days post initial activation).

### Transduction of CD4+ primary T cells and infection with HIV

Human CD4+ cells were isolated from naïve PBMCs using CD4+ T cells isolation kit (RosetteSep), followed by centrifugation via density gradient medium. CD4+ T cells were stimulated with CD3/CD28 beads for 48 hr. and then transduced with HA-CycT1-V107E or HA-CycT1 wild type expressing lentivirus. 48 hr. post transduction, cells were infected with HIV-LTR-Tat-BFP lentivirus at an MOI of 1. As control cells were also transduced with a CMV-BFP infectious lentivirus. Viral replication was measured by FACS.

### RNA Precipitation

HEK-293T cells were grown on 10 cm plates and were co-transfected with 10 μg of pHAGE-HA-CycT1 mutants (or HA-CycT1-wild type), 5 μg of HIV LTR-Luc, 5 μg pCDNA-CMV-Myc-Tat and 2 μg of pCDNA-CMV-GFP. 48 hr. post transfection, 90% of cells expressed GFP, indicating high transfection efficiency. Cells were lysed with 800 μl of RNA-IP buffer, supplemented with RNAse inhibitors (NEB) and protease inhibitors (Lysis buffer containing; 0.5% NP-40; 20 mM HEPES pH 7.8; 100 mM KCl; 0.2 mM EDTA; PI cocktail Sigma (add fresh before use, 1:100); RNAse inhibitor (NEB). Cell lysates were incubated on ice for 10 min. and centrifuged at 5000Xg for 5 min at 4°C. Supernatants were collected and then divided into two aliquots. The first was incubated overnight with gentle rocking with 1 μg of anti-HA antibody (ab-9110), and the second was incubated with control non-immune anti-human IgG. Next, protein A-Sepharose beads were pre-blocked with BSA and yeast tRNA and then were added for additional 2 hr. at 4°C. 50 μl of cell lysate was collected for input and stored at −80°C till RNA extraction. Beads were then extensively washed with washing buffer containing; 0.1% NP-40; 20 mM HEPES pH 7.8; 100 mM KCl; 0.2 mM EDTA; PI cocktail Sigma (add fresh before use, 1:100); RNAse inhibitor (NEB). Washing was performed for 10 min with gentle rocking at 4°C, while eppendorf tubes were replaced at least once to avoid unspecific RNA binding to the tubes. Following wash, beads were centrifuged 3000 rpm for 3 min. Beads were re-suspended in 100 μl of lysis buffer and extracted with 100 μl Tris-Phenol and chloroform followed by ethanol precipitation. Input samples were extracted using the same protocol. RNA concentrations were measured and 1 μg was used for the synthesis of cDNA, using cDNA high capacity kit (Applied Bio-system) in a total volume of 20 μl. Inputs were re-suspended in 20 μl of DEPC water as well. IP RNA was then subjected to cDNA synthesis, which was further analyzed by real-time PCR analysis using KAPA SYBER GREEN fast mix with TAR and 7SK snRNA specific primers. 2 μl of 1:10 and 1:100 dilution of input were used to generate a standard curve and determine the quality control of the reaction. 2 μl of undiluted IP samples were used as templates. Reactions were analyzed in triplicates and presented as fold of enrichment relatively to PCR results obtained for cells transfected with LTR-Luc alone - set to 1.

## Competing interests

The authors declare that they have no competing interests.

## Authors’ contributions

AK, MM and SG performed the described experiments. AK and RT conceived and designed the experiments; NV and OB provided the CycT1 mutants, which were sub-cloned into the lentivector by AK. OB shared discussions regarding experimental design and structure implications. All authors read and approved the final manuscript.
